# The Current Landscape of Modular CAR T Cells

**DOI:** 10.3390/ijms262411898

**Published:** 2025-12-10

**Authors:** Alexander Haide Joechner, Melanie Mach, Ziduo Li

**Affiliations:** 1 Biosceptre (Aust) Pty Ltd., Westmead, NSW 2145, Australia; alex.joechner@biosceptre.com (A.H.J.); melanie.mach@biosceptre.com (M.M.); 2 Sydney Medical School, Faculty of Medicine and Health, University of Sydney, Sydney, NSW 2006, Australia; 3 St George and Sutherland Clinical School, Faculty of Medicine and Health, UNSW, Sydney, NSW 2052, Australia

**Keywords:** antigen receptor, CAR T cell, adaptor CAR, modular CAR, switchable CAR, indirect CAR, adaptor molecule (AM)

## Abstract

Despite the groundbreaking impact of currently approved CAR T-cell therapies, substantial unmet clinical needs remain. This highlights the need for CAR T treatments that are easier to tune, combine, and program with logic rules, in oncology and autoimmunity. Modular CAR T cells use a two-part system: the CAR on the T cell binds an adaptor molecule (AM), and that adaptor binds the tumour-associated antigen (TAA). This design separates recognition of the target antigen and activation of the T cells, resulting in a cellular therapy concept with better control, flexibility, and safety compared to established direct-targeting CAR T-cell systems. The key advantage of the system is the adaptor molecule, often an antibody-based reagent, that targets the TAA. Adaptors can be swapped or combined without re-engineering the T cells, enabling straightforward multiplexing and logic-gated control. The CAR itself is designed to recognise the AM via a unique tag on the adaptor. Only when the CAR, AM, and antigen-positive target cell assemble correctly is T-cell effector function activated, leading to cancer cell lysis. This two-component system has several features that need to be considered when designing a modular CAR: First, the architecture of the CAR, i.e., how the binding domain and the backbone are designed, can influence tonic signalling and activation/exhaustion parameters. Second, the affinity of CAR–AM and AM–TAA will mostly define the engagement kinetics of the system. Third, the valency of the AM has an impact on exhaustion and non-specific activation of CAR T cells. And lastly, the architecture of the AM, especially the size, defines the pharmacokinetics and, consequently, the dosing scheme of the AM. The research conducted on direct-targeting CAR T cells have generated in-depth knowledge of the advantages and disadvantages of the technology in its current form, with remarkable clinical success in relapsed/refractory disease and long-term survival in otherwise difficult-to-treat patient populations. On the other hand, CAR T-cell therapy poses the risk of severe adverse events and antigen loss coupled with antigen-negative relapse which remains the main reason for failed therapies. Addressing these issues in the traditional setting of one CAR targeting one antigen will always be difficult due to the heterogeneous nature of most oncologic diseases, but the flexibility to change target antigens and the modulation of CAR T response by dosing the AM in a modular CAR system might be pivotal to mitigate these hurdles of direct CAR T cells. Since the first conception of modular CARs in 2012, there have been more than 30 constructs published, and some of those have been translated into phase I/II clinical trials with early signs of success, but whether these will progress into a late-stage clinical trial and gain regulatory approval remains to be seen.

## 1. Introduction

Chimeric antigen receptor (CAR) T-cell therapy has cemented its position in the treatment of B-lineage leukemic disease such as ALL and B-cell lymphomas since the pivotal studies [1] leading to the approval of Tisagenlecleucel (Kymriah^®^). Today, there are seven approved CAR T-cell therapies for the treatment of B-cell acute lymphoblastic leukaemia (B-ALL) and Multiple Myeloma (MM) [2,3].

Building on these clinical and regulatory approvals, several next-generation strategies are now entering the clinic, including armoured CAR T cells (e.g., IL-18-secreting huCART19-IL18 which rescue patients after the failure of standard CD19 CAR-T [4], direct in vivo CAR-T engineering which simplifies the clinical application complexity [5], solid-tumour CAR T therapies with clinically meaningful efficacy such as CLDN18.2-directed CAR-T cells in gastroesophageal cancer [6], and the extension of CAR T-cell therapy to refractory autoimmune diseases through resetting the immune cells [7]. For a more comprehensive summary from a translational perspective on CAR T-cell therapy in cancer, the recently published article by Brudno et al. is highly recommended [8]. Despite the success of these CAR T cells in improving treatment outcomes, there are barriers to broadening the clinical application of CAR T cells beyond the currently approved indications to other cancer entities such as acute myeloid leukaemia (AML) and solid tumours. The conventional CAR-T design faces multiple challenges, including uncontrolled activation, disease heterogeneity, antigen escape, and a hostile tumour microenvironment when treating these diseases [9]. To solve these challenges, the next step in CAR T-cell design is urgently needed.

The modular CAR system had its first conceptualisation in the early 2000s [10]. The core principle of modular CAR systems is the decoupling of antigen recognition and CAR-mediated T-cell activation via an adaptor molecule (AM). Dividing those two functions enhances versatility by modular targeting and allows for CAR T-cell modulation through AM dosing to improve safety and efficacy.

Direct CAR constructs lack a mechanism to control the activation level, unless specifically engineered with a safety or suicide switch to shut down CAR signalling. Modular CARs, on the other hand, can be switched “on” and “off” intermittently, giving T cells a break and reducing the impact of overactivation or treatment-related toxicities such as cytokine release syndrome (CRS) and Immune Effector Cell-Associated Neurotoxicity Syndrome (ICANS). The AM is based on therapeutic antibodies (Ab), which enables straightforward logical combination of targeted antigens in case of tumour heterogeneity and antigen-escape. Notable challenges with this technology include the increased complexity, as it functions as a two-component system with distinct pharmacokinetics (PK) and pharmacodynamics (PD), the associated rise in production costs, and the regulatory challenges for approval of a dual-component cellular product compared to a direct targeting CAR.

**Figure 1:**
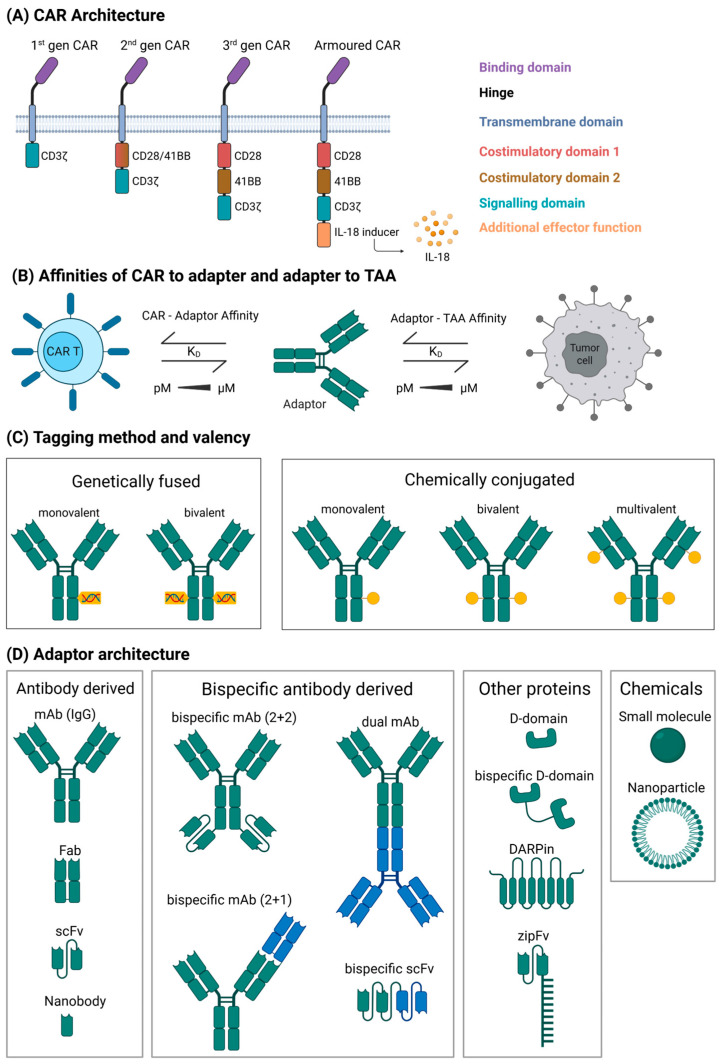
Key considerations for modular CAR design. The key aspects to consider when designing a modular CAR are as follows: (**A**) architecture of the CAR, (**B**) affinity of CAR to tag and AM to TAA, (**C**) valency of the AM and how to incorporate the tag into the structure, and (**D**) the architecture of the AM. (**A**) CAR constructs from first to third generation will differ in the costimulatory domains included [13], whilst the other components (hinge, transmembrane (TM), and signalling domains) can vary. Most important is the binding domain or extracellular domain (ECD) as this will define the interaction with the AM; most constructs will have an antibody fragment as a binding domain, but other proteins have been shown to work as effectively (CD16, protein G, etc.). Armoured CAR T cells include an additional module that usually delivers cytokine support. Here, we have chosen to show an IL-18 secreting module. (**B**) Affinity dictates the kinetics of CAR engagement; in the modular CAR system, there are two affinities that can be varied: between CAR and the tag on the AM and between AM and TAA. Both typically range from low pM to µM with the CAR—Tag affinity being more important for the CAR engagement since the AM–TAA affinity is mostly dependent on the antibody clone used to target the TAA. (**C**) The valency of the AM strictly depends on the number of tags, which can be introduced either genetically fused to the AM or chemically conjugated. Genetic fusion allows greater control of tag numbers, whereas chemical conjugation is simpler but typically yields in a random number of conjugations averaged between all molecules in a batch. (**D**) The format of the AM is critical for pharmacokinetics and dosing: smaller molecules are easier to control but require constant application and monitoring, while larger molecules will remain in the system for longer. The majority of AMs used are antibody- or peptide-derived but a small number of researchers are investigating other formats, such as small molecules or lipid nanoparticles. Created in BioRender. Joechner, A. (2026) https://BioRender.com/3kus741 (accessed on 27 November 2025).

The key design attributes of a modular CAR T platform comprise (1) the CAR constructs, (2) the coupling mechanism and affinity between the CAR binder and the AM molecule, (3) AM modification/tagging technology and the valency of the tags, and (4) the antibody formats of the AM molecules. Figure 1 provides an overview of the key aspects to consider when designing a modular CAR system based on the constructs presented in this review.

Alongside the broader progress in CAR design, the landscape of modular CAR platforms has evolved substantially with innovative strategies to improve functionality and specificity. These advances include CAR construct delivery and manufacturing methods [11] and improved insight into CAR T-cell therapy in humans, which is equally applicable to modular CAR systems. Furthermore, advances in bi-specific antibody and T-cell engager technologies have deepened the understanding of multi-targeting strategies and immune cell-engagement logics, providing valuable insight for the multi-specific modular CAR targeting strategy and CAR T-cell recruitment, activation, and modulation [12].

This review explores the differences between modular CAR platforms in the literature, focusing on systems that have been or are currently being translated into clinical trials.

## 2. Modular CAR Terminology and Category

In this review, the term modular CAR is interchangeable with indirect CAR, adaptor CAR or split CAR. While some authors also refer to these as universal CARs, we consider the term universal CAR more appropriate for off-the-shelf products derived from allogeneic T cells, which can be manufactured in advance and used without further processing [14]. As mentioned before, the modular CAR requires an AM to recruit CAR-expressing cells, and according to the technology of this coupling mechanism, we can divide the modular CAR systems into certain categories:

- **Peptide tags**: These tags are usually small peptides genetically fused to the AM and a corresponding antibody-derived binding domain on the CAR. Examples are the UniCAR [15] recognising a peptide derived from the La/SS-B autoantigen or the PNE CAR [16] recognising a yeast transcription factor-derived tag. A variation of this system has a binding domain and protein epitope switched so that the CAR expresses the tag and the AM contains the binding domain.
- **Chemical tags**: These tags are similar to peptide tags but the tag is a chemical conjugated to the AM, and which is recognised by the CAR. Examples are FITC-derived [17,18] or biotin-derived [19,20,21] systems.
- **Ab domain recognition**: A different strategy for designing modular CAR systems is to use CAR T cells specific to antibody domains, mainly the Fc domain in the context of full-size antibodies or antibody fragments. Examples are CD16-derived CARs that bind Fc domains [22,23], the P329G mutated Fc-domain with a CAR specific to this mutation [24], artificial epitopes (“meditopes”) inserted between light and heavy chains [25], or a protein G-derived CAR-AM combination [26].
- **Covalent bond**: This type of interaction relies on the formation of a covalent bond between CAR and AM after coupling, resulting in a strong activation signal but less flexibility to swap out the AM. The systems in this category use bacterial or other proteins capable of spontaneously forming covalent bonds [27,28,29].
- **Engineered protein pairs**: This category includes modular CAR systems that are derived from the receptor–ligand type of protein pairs engineered for improved binding. These are mostly derived from natural receptor–ligand pairs such as leucine zippers [30], Bim/Bcl-2 [31], or NKG2D/ULBP2 [32].

Table 1 gives an overview of the constructs discussed in this review.

## 3. Landscape of Modular CAR Constructs

### 3.1. UniCAR

The UniCAR platform, first reported by the Bachmann group in 2016, redirects CAR specificity to a 10-amino-acid (AA) epitope (“5B9” tag) derived from the La/SS-B nuclear autoantigen, located between the N-terminal La motif and the first RNP consensus sequence. Expression of this motif is minimal under physiological conditions but is elevated on dying cells and in autoimmune diseases such as systemic lupus erythematosus (SLE) and Sjögren’s syndrome (SS) [15,48]. The CAR construct comprises a single-chain variable fragment (scFv) derived from the anti-La antibody 5B9 followed by a CD28 hinge and transmembrane (TM) domain and CD28/CD3-ζ intracellular signalling domain. The AM consisting of small antibody fragments and their derivatives recognising a tumour antigen; each AM is genetically fused with the 5B9 tag [15,33]. This design has a low nanomolar affinity of CAR to AM with EC_50_ values in the low picomolar range [49]. The proof-of-concept study for the UniCAR system was conducted in AML indications targeting CD33, CD123, and later for the B-lineage antigen CD19 and common solid cancer targets of EGFR, PSMA, and PSCA [33,50,51]. The preclinical data demonstrated that the modular CAR system is tuneable, easy to control, and has good efficacy in vivo, which led to the initiation of two clinical trials: NCT04230265 targeting CD123 in AML patients and NCT04633148 using PSMA as a target for prostate cancer. Initial reports from the AML trial demonstrated a clinical response in 15 patients after receiving the CAR T product with good safety profiles, including one patient who achieved CR adverse events, including grade I and II CRS and grade II ICANS, resolved within 24 hrs of stopping treatment with their AM [52].

A variant of the UniCAR, termed RevCAR, relocates the 5B9 tag to the CAR extracellular domain (ECD) and fuses the anti-La scFv to the AM. This inversion reduces the CAR construct size for lentiviral transduction and enables the addition of other effector functions, e.g., dual CAR T function for logic gating [37,50]. The RevCAR is being evaluated in an ongoing phase I trial (NCT05949125) for AML, using the CD123 AM similar to the UniCAR in the setting of allogeneic CAR, developed with CRISPR/Cas9. The trial commenced in 2024 with results not yet reported.

### 3.2. PNE-CAR

The PNE-CAR system, first described in 2016 by Young et al. [16], employs a modular CAR system in which CAR receptor engagement is mediated by a peptide neo epitope (PNE), a 14 AA sequence derived from the yeast transcription factor GCN4. The PNE is genetically fused to a Fab-format adaptor molecule specific for tumour recognition. The CAR recognising the tag contains an scFv with high affinity (K_D_: 5.2 pM) [53] to the PNE followed by CD8A hinge and TM, 4-1BB costimulatory, and CD3ζ signalling domains [16,54]. Because the GCN4-derived tag is absent from human cells, the CAR on its own is functionally inert. This peptide-specific switchable CAR T cell (sCAR-T) showed efficacy in preclinical proof-of-concept studies targeting CD19, CD20, or Her2 [54], demonstrating that the CAR T-cell activation is dose-dependent as well as the flexibility of switching between different antigens in vitro and in vivo.

Clinical evaluations are underway for CD19-positive relapsed/refractory B-cell malignancies (NCT04450069) and Her2-positive breast cancer (NCT06878248). The CD19 preliminary report observed no dose-limiting toxicities [55] with a 78% objective response rate (ORR) and a 67% complete response (CR) in nine patients [56]; however, the final results are pending. The Her2 trial is currently recruiting. With the growing strategy of using CAR T cells to reset the pathogenic humoral immune responses in autoimmune disease, a clinical trial of CD19-targeting sCAR T cells has been registered and is currently recruiting (NCT06913608).

In 2021, Qi et al. reported a cp-Fab/CAR-T platform [35] derived from the PNE-CAR. It uses the same CAR and peptide tag but the key difference is that the adaptor Fab is based on the catalytic antibody h38C2, which harbours an unprotonated lysine residue [57] that enables site-specific conjugation with small molecules or chemical agents. In this case, the Fab itself is non-reactive; however, when conjugated with folate, it binds to the folate receptor 1/alpha (FOLR1/FRα) which can be upregulated on tumour cells [58]. This approach renders the Fab itself obsolete as a targeting element, raising the question of whether the PNE tag could instead be directly conjugated to the small molecule, thereby potentially leveraging the improved tissue penetration offered by this design.

### 3.3. Other Peptide-Based CARs

The **SAR T-cell**, synthetic antigen receptor T-cell, is constructed from the extracellular domain of either EGFRv3 or human Crypto-1 fused to the CD28 and CD3ζ domains. The AMs for SAR T cells are bi-specific antibodies targeting EpCAM or mesothelin linked to an EGFR/Crypto-1 targeting scFv to recruit effector cells. This system could elicit a strong anti-tumour response in vitro and in vivo against EpCAM- and mesothelin-expressing cells in a dose-dependent manner. Interestingly, the authors found that a monovalent AM improves off-tumour toxicity and tonic signalling compared to the bivalent AM. Beyond that, regular cetuximab could be used to eliminate SAR T cells from circulation, showcasing the tuneability, control, and safety of this system [36].

The **Fabrack-CAR** designed by Kuo et al. [25] is built around the “meditope” developed in their lab, a cyclic, 12-amino-acid-long peptide that can specifically bind to a pocket between the heavy and light chain of cetuximab which can also be engineered into other monoclonal antibodies [59,60]. This meditope serves as the ECD for the Fabrack-CAR together with a linker (PAS or PAS-CH3), CD28, CD28, or 41BB costimulatory domains, and CD3ζ signalling domain. Full-sized antibodies or Fab fragments enabling binding of the meditope (memAb or meFab) are used as AMs targeted towards Her2, EGFR, CD33, Cadherin-6 (CDH6), or CD20. Together with these AMs, the Fabrack-CAR could lyse antigen-positive tumour cells in a dose-dependent manner and multiple antigens could be targeted sequentially or in parallel [25], demonstrating the functionality, control, and versatility of the system.

The **conduit CAR** concept leverages a common sequence on CAR T cells, the G4S linker, as a recruitment tag for an AM that redirects CAR T-cell specificity. The authors generated GLPB30, an anti-G4S antibody with low nanomolar affinity for G4S, and formatted it as a bi-specific AM. The N-terminus recognises the TAA and the anti-G4S scFv is fused to the IgG C-terminus, or vice versa. To prevent self-binding, AM constructs use a non-G4S linker for the scFv at the C-terminus, specifically a modified Bird et al. linker [61]. The CAR itself, containing a human germline antibody 3-23/B3-derived scFv is inert on its own but paired with AM targeting PSMA or TMEFF2 (tomoregulin-2) it enabled dose-dependent tumour lysis and cytokine secretion. Interestingly, the authors could also demonstrate the functionality of this system using the well-established CD19 CAR to target a CD19-negative cell line [38]; however, no in vivo validation was reported.

Aleta Biotherapeutics developed a **CD19 CAR engager molecule** compatible with existing CD19 CAR T therapies first reported in 2019 [45,62,63,64,65]. Their studies used an FMC63 anti-CD19 CAR in a third-generation backbone. The engager comprises a CD19 ectodomain fused to scFv or VHH against diverse TAAs to retarget CD19 CAR T cells: EGFR, HER2, bi-specific Her2/EGFR, CLEC12A, CD33, bi-specific CD33/CLEC12A, or CD20 [45,62,63,64,65]. The usage of CD19 ECD to redirect CD19-targeting CAR T cells is a unique feature of this system, enabling the sequential treatment of CD19-positive diseases. Phase I and II clinical trials are underway in patients previously treated with anti-CD19 CAR T cells (NCT06045910), with results not yet reported.

The **TRUE CAR** system uses fusogenic antigen-loaded nanoparticles (F-AgNPs) with EGFRvIII-derived peptide to redirect CAR T cells [39]. The nanoparticles, which are loaded with an EGFRvIII-derived peptide, can be inserted into the tumour membrane and are recognised by an EGFR targeting CAR. Tumour cells labelled with F-AgNPs induced CAR T-mediated cytotoxicity and reduced viability in a tumour spheroid model. Preferential integration of the nanoparticle into tumour cells was observed, though the mechanism remains unclear [39].

### 3.4. Biotin-Based CAR

Biotin-based modular CAR T cells are some of the earliest designs in the adaptor CAR field. As biotin conjugation is simple, fast, and reliable, developing CAR T cells that recognise biotinylated adaptors is straightforward. Urbanska et al. introduced the **biotin-binding immune receptor (BBIR)** in 2012 [21], using avidin linked to a second-generation CAR construct. Biotinylated scFv or IgG1 adaptors targeted EpCAM, FRα, or mesothelin-bound avidin with a K_D_ of around 10^−7^ M and redirected the CAR against ovarian cancer cell lines, but the approach did not progress to clinical testing [21]. In 2014, the same group published a different version of their modular CAR that replaced the biotin–avidin with an FRα-FRα scFv, with FRα as the ECD to engage with the adaptor [42]. Similarly, in 2017, Lohmueller et al. [20] reported their modular CAR using an affinity-enhanced **monomeric streptavidin 2 (mSA2)** biotin-binding domain, with higher biotin affinity (K_D_ = 5.5 × 10^−9^ M) which enabled a biotinylated IgG1 AM targeting CD19 or CD20 to elicit potent activity in vitro, although no in vivo data was presented. Kourtesakis et al. expanded the mSA2 modular CAR to treat the glioblastoma-targeting antigens CD276, EPHA2, CD70, and IL13Ra2. CAR T cells slowed tumour growth in an orthotopic PDX model when combined with the AM, which aligned with the significant upregulation of T-cell activation markers reported [66]. To our knowledge, this is the first modular CAR platform tailored specifically to brain tumours, demonstrating the promise of this technology.

The leading biotin-based modular CAR is the AdCAR, developed by Seitz et al. with Miltenyi’s support and first reported in 2021 [19]. It uses the mBio3 antibody, which recognises a “linker–label epitope” (LLE) between biotin and an LC-LC-NHS linker. The CAR uses an mBio3-derived scFv while the adaptors are full-sized mAbs or Fab fragments biotinylated with this specific linker. The system showed activity on CD19 and GD2 models [19] and was later expanded to the AML targets CD33, CD38, CD123, CD135, and CD371 (CLL-1) [67], suggesting tuneable, multiplexed, and logic-gating strategies, though clinical validation is still lacking. Building on this, the AdCAR concept extends to an off-the-shelf CAR-NK system, exemplified by the NK-92-based AdCAR effector cells that showed in vitro activity against CD19/CD20 [68] and solid cancer antigens such as EGFR and CD276 [69].

### 3.5. FITC-Based CAR

FITC (fluorescein isothiocyanate) was used as one of the earliest tags together with biotin for modular CAR systems. Several groups have published constructs using FITC-tagged AM and an anti-FITC CAR [17,18,40,41]. In the antibody and antibody fragment modality, Young’s group not only created the PNE CAR [16], but also designed a second-generation CAR using an anti-FITC scFv (clone 4m5.3), achieving low nanomolar affinity between CAR and FITC. FITC-tagged Fab fragments targeting CD19 had an in vivo efficacy comparable to a FMC63-based CD19 CAR [17].

A different approach was pursued by the Low group in the small-molecule modality. Their CAR incorporated the same anti-FITC clone 4m5.3 (K_D_ = 30 pM) or the humanised version E2 (K_D_ = 0.75 nM) in a second-gen CAR backbone with different hinges [18,40]. The pairing AMs are FITC-conjugated small molecules including FITC-folate recognising the folate receptor, FITC-DUPA binding to PSMA, and FITC-AZA binding to CA IX (carbonic anhydrase IX). These AM were able to induce potent cellular cytotoxicity, significantly hindering tumour growth in vitro and in vivo [18]. The FITC-folate version was also investigated on paediatric solid tumours, demonstrating control, safety, and potent anti-tumour activity [40]. No FITC-CAR clinical trials have been registered to date.

Building on the 4m5.3 antibody and a FITC tag, Zhang et al. in 2023 explored a PEG–lipid–FITC amphiphile delivered by intratumoral injection, paired with a second-generation CAR [41]. The AM, which is a FITC–PEG–lipid conjugate termed “Amph-FITC”, can insert itself into the cell membrane of tumour cells, wielding in vitro cytotoxicity against melanoma cells and in vivo CAR T-cell expansion, anti-tumour activity, and improved survival [41]. The non-specific membrane insertion poses off-tumour toxicity risk, and the feasibility of this approach remains to be validated in clinical trials.

### 3.6. CD16-Based CAR

The concept of harnessing the mechanism of antibody-dependent cellular cytotoxicity (ADCC) by using the physiological affinity of CD16 for immunoglobulins and expanding it to T-cell-based therapies is well established. Clemenceau et al. first reported it in 2006 [33], engineering T cells with a CD16/γ chimeric receptor to elicit ADCC against CD20-positive target cells in the presence of rituximab. This concept was applied to CAR T cells by a few different groups, including Yasukawa [43,70] and Campana [22] in 2014, D’Aloia et al. in 2016 [44], and the Kobold group in 2019 [23].

Using a first-generation CAR, Yasukawa et al. fused the CD16 ECD and a CD3ζ TM domain and signalling module, generating modified T cells that responded to cancer cells labelled with antibodies targeting CD20, HER2, or CCR4 in vitro and in vivo [43,70]. Because native CD16 binds IgG weakly, first-generation CD16–CD3ζ CAR T cells showed limited potency. Campana et al. addressed this with a high-affinity F158V polymorphism of CD16 (K_D_ = µM [71]) as an ectodomain followed by a second-generation CAR design with a 41BB intracellular domain, dubbed the **antibody-coupled T-cell receptor (ACTR)**. They demonstrated in vitro and in vivo efficacy against CD20, Her2, or GD2 through ADCC-mediated CAR T-cell cytotoxicity. These findings led to clinical trials in CD20-positive lymphoma and HER2-positive solid tumour malignancies (NCT02776813 and NCT03189836, NCT03680560). The CD20 studies showed meaningful responses with manageable toxicity [72,73,74], whereas the HER2 trial terminated early with no data disclosed [75]. There are also reports of other researchers using this strategy, but with a CD28 costimulatory domain [44]. Finally, the Kobold lab’s ACTR used the high-affinity CD16 158V ectodomain with a standard second-generation CAR construct to target EGFR, CD20, MSCP adaptor-labelled cells. Notably, they showed that ADCC potency depends on both tuning CD16 affinity and Fc glycoengineering of the antibodies, though no in vivo data was provided [23].

### 3.7. Antibody Domain Recognising CAR

These are antibody-based modular CARs that recognise the constant region of the AM without requiring a peptide or chemical tag. This class includes the P329G-targeting CAR [24] and GA1 CAR [26].

The **P329G CAR T cell** recognises the P329G mutation of P329G and L234A/L235A (PG-LALA) mutated Ig which lacks FcγR engagement [76]. The second generation of P329G CAR with the CD28 costimulatory domains has been used to target mesothelin, HER2 [76], CLND18.2 [77], and the AML antigens CD33 and CD123 [78]. A notable feature is the steric hindrance: the CAR can only engage with one of the two P329G mutations present in the CH2 domain, which prevents unspecific CAR dimerization and activation [24], an issue for many modular CAR platforms. Phase I clinical trials for the treatment of CLND18.2-positive [77] (NCT05199519) and BCMA-positive [79] (NCT05270928, NCT05266768) cancers report a manageable safety profile and early clinical responses, encouraging further investigation [77,79].

The **GA1 CAR** reported by Arina et al. [26] utilises the protein G variant GA1 as the CAR docking domain in a second-generation backbone with the 41BB costimulatory domain. Based on protein G interacting with the constant light chain (C_L_) domains of antibodies or Fab fragments [80], GA1 is engineered to recognise mutated Fab scaffolds (LRT/SQRT) with high affinity (K_D_ 100 pM–10 nM) while sparing wild-type light chains, guaranteeing system specificity. The proof-of-concept study demonstrated the dose- and affinity-dependent in vitro and in vivo functionality of the GA1 CAR, targeting EGFR or HER2-positive cancer cells. Interestingly, exposure of GA1 CAR T cells to the adaptor molecule alone introduced mRNA-level activation signature upregulation without substantial upregulation of surface activation markers.

### 3.8. Covalent Bond

Covalent modular CARs couple the AM with the CAR through an irreversible bond to mimic the stability of direct CAR T cells while still retaining the flexibility to switch targets. The covalent modular CAR constructs include the SpyCatcher–SpyTag, barnase–barstar, and SNAPtag–benzylguanine (BG) systems [27,28,29,46].

**OmniCAR**, the most prominent covalent CAR developed by Minutolo et al. [29], was built on the SpyCatcher–SpyTag chemistry that was developed a decade earlier [81]. This system relies on a spontaneous covalent bond formed between two proteins derived from the *S. pyogenes* fibronectin-binding protein FbaB: SpyCatcher (138AA) and SpyTag (13AA). OmniCAR employs a truncated SpyCatcher ectodomain paired with SpyTag-equipped antibodies or designed ankyrin repeat proteins (DARPins), demonstrating in vivo efficacy targeting CD20, Her2, EpCAM, or EGFR tumours [29,82]. Chen et al. later introduced an optimised variant of the SpyCatcher–SpyTag system termed SDCatcher/GvOptiTag (Sd/Gv) in a third-generation CAR backbone. The authors included a CRISPR/Cas9-mediated TCR and HLA-I knockout in their CAR T generation process to establish a donor-independent CAR T-cell system and scFvs containing the GvOptiTag as an AM targeting the HIV-1 precursor glycoprotein gp160 (clone VRC01) or a bi-specific AM targeting CD5 and CD30. These **modular universal CAR T (MU-CAR-T)** cells demonstrated potent anti-tumour activity and were also able to suppress HIV-1 rebound after cessation of antiviral treatment in vitro [28]. However, bacterial origin immunogenicity and the impact on switch retargeting by the covalent binding remain concerns. No clinical trials are registered yet.

Stepanov et al. (2022) described a **barnase/barstar-based CAR (bsCAR)** [27]. The barnase and barstar pair has sub-picomolar affinity (10^−14^–10^−13^ M) [75], making this noncovalent system akin to covalent modular CARs. The bsCAR uses a mutated barstar as an ECD preventing homodimerization and a second-generation backbone. Barnase-tagged DARPins targeting HER2/EpCAM induced dose-dependent cytotoxicity, cytokine release, and in vivo tumour clearance. Interestingly, owing to the RNA-toxic nature of barnase, the authors observed an anti-tumour effect with only the AM after a prolonged incubation time (10 d) in vitro but not in vivo [27].

Extending on their biotin modular CAR work [20], the Lohmueller group developed the **SNAP-CAR** [46], whose SNAPtag ectodomain covalently binds benzylguanine (BG) to capture the BG-conjugated adaptors [83]. BG-conjugated antibodies or fragments against CD20, EGFR, or HER2 activated CAR T cells in vitro and inhibited tumour growth in an HER2 xenograft model in vivo. Notably, the SNAP-CAR required lower AM doses than the mSA2-CAR, likely due to covalent engagement, and untargeted O6-BG can be applied as a competitive inhibitor to improve safety.

### 3.9. Engineered Protein Pair CAR

This section summarises modular CAR concepts using engineered protein pairs, including leucine zipper-based, NKG2D/ULBP interaction, BCL-2/BIM interaction, and AFP-derived D-domains [30,31,32,47].

Cho et al. introduced the **SUPRA-CAR**, a split, universal, programmable platform with a leucine zipper as a CAR ectodomain paired with a cognate zipper-tagged scFv-based AM targeting Her2, AxL, or mesothelin. The authors could show that the affinity between CAR and AM influences cellular cytotoxicity, the potential of logic gating with different AM targets, and in vivo tumour clearance. They further demonstrated a true AND gate configuration, where separate CAR T cells bearing different CD28 or 41BB costimulatory regions signalled only when both bound to two distinct antigens on the same target cell via the AM [30].

Logic gating is the main pillar that sets modular CAR concepts apart from traditional ones. In contrast to the SUPRA-CAR logic-gating strategy, which separates costimulatory domains, the **CO-LOCKR CAR** sets the logic gates at the adaptor level using a “cage–latch–key” switch [31]. A LOCKR-based, three-component design involves a cage that hides a Bim latch and a key that reveals it. Both are DARPin-fused to distinct TAAs (HER2, EGFR, and EpCAM), so the cage and key must co-localise on the tumour cell surface. The exposed Bim recruits its ligand, the BCL-2 ectodomain on CAR T cells, and triggers antigen-dependent cytotoxicity and cytokine release [84]. Notably, the system demonstrated robust AND, OR, and NOT gating [31]. While in vivo validation is needed, this level of logic gating confers a unique advantage of the CO-LOCKER CAR among modular CAR T-cell platforms.

The **convertibleCAR** platform [32] developed by Xyphos (Astellas) employs the NKG2D and ULBP interaction to retarget CAR T cells. A mutated, inert NKG2D variant (iNKG2D) serves as the ECD for the CAR and engages with a ULBP2-based peptide (U2S3) on the adaptor, called “MicAbody”, to retarget CAR T cells. HER2- or CD20-specific MicAbodies demonstrated dose-dependent cytotoxicity and control of disseminated or subcutaneous xenograft models. Interestingly, they also demonstrated the capability of the platform to deliver cytokines such as mutated IL-2 or IL-15 to T cells. This data led to initiation of a phase I CD20 clinical trial (NCT06248086), which was terminated later without outcomes reported.

Arcellx’s **ARC-SparX** system [47] is based on the human alpha-fetoprotein (AFP)-derived tag D-domain binder. The D-domain is a 73 AA synthetic protein that can be used as an alternative binding domain to an antibody [85,86,87]. The antigen-receptor X-linker protein (SparX) is an AFP-tagged mono- or bivalent D-domain AM that redirects CAR T cells towards the antigen-positive target cells with high affinity (K_D_ = 1.8 nM). TAAs, BCMA and CD123, were validated preclinically [47,85,86,88] followed by the two phase I clinical trials (NCT05457010, NCT04155749) with results to be disclosed.

## 4. Clinical Landscape

Early-phase clinical trials indicate that modular CAR T cells are generally well tolerated with manageable toxicities that subside upon cessation of the AM, while maintaining anti-tumour activity. At this stage, most clinical programs remain in phase I and are actively recruiting, with further data yet to be reported. Despite early signs of success in these trials, it remains uncertain whether these programs will advance to later-stage clinical trials and eventual regulatory approval. Nonetheless, across solid tumour applications (HER2, GD2, PSMA, and CD276), feasibility and proof of concept have been established; however, tumour heterogeneity and the suppressive tumour microenvironment remain major barriers to efficacy. All clinical trials investigating modular CAR T cells are listed in Table 2.

## 5. Key Design Factors for Modular CAR Systems

The landscape of modular CAR T-cell systems has grown substantially since the concept first emerged in 2012, and today there are many different approaches. All are unified in the goal of making CAR T-cell therapy more flexible and safer with the addition of the AM, which can be easily controlled, switched, or blocked. There are a few key attributes that need to be considered when designing a modular CAR construct: affinity, AM format and pharmacokinetics, logic-gating strategy, and tonic signalling, as well as immunogenicity and toxicity.

### 5.1. Affinity

The affinity between the CAR and recruiting AM is a key determinant of CAR activity. If it is too high, CAR T cells are prone to exhaustion and on-target, off-tumour toxicity; if too low, engagement with targets expressing low antigen density becomes inefficient. Whether higher- or lower-affinity CARs are preferable remains unresolved and likely depends on the target antigen and the CAR architecture [90,91,92]. The affinity of modular CARs to the AM discussed in this review ranges from pM to nM, comparable to conventional direct-targeting CARs except for covalent systems such as the OmniCAR, MU-CAR, bsCAR, or SNAP-CAR. However, only focusing on the equilibrium affinity (K_D_) can be misleading since it is defined as the ratio of the binding kinetics of association (K_on_) and disassociation (K_off_) rate which means they can differ significantly at the same K_D_. The optimal affinity for CAR T engagement will require a fast K_on_ whilst K_off_ can be more variable so that CAR T cells can recognise cancer cells and become activated without dissociating from the target cells too quickly. This will generally lead to the affinity range mentioned above, confirmed by the clinically tested PNE-CAR (K_D_: pM) or UniCAR (K_D_: nM). The ACTR utilises high-affinity F158V polymorphism of CD16 (K_D_ = µM) which is considerably lower than the other two and that might contribute to the reduced clinical efficacy.

Covalent CAR systems behave similarly to direct CARs after the formation of AM–CAR complexes, which have a robust response upon cancer encounter. However, switching antigens and exerting temporal control over activation becomes dependent on receptor turnover, limiting the adaptability of this technology.

### 5.2. Tonic Signalling

A related consideration is tonic signalling, which refers to the baseline activation of CAR T cells in the absence of a target antigen. The prevailing view has been that tonic signalling is detrimental in CAR T cells as it leads to exhaustion and impairs anti-tumour activity [93,94]. Recent work, however, supports the idea that low levels of tonic signalling can be beneficial for CAR T cells, enhancing proliferation, longevity, and functional readiness [91,95,96,97], and our own observations align with this hypothesis. This is biologically plausible because tonic signalling is not unique to genetically modified T cells but rather a physiological feature of unmodified lymphocytes [98]. These insights are particularly relevant for modular CAR platforms, where antigen engagement is intermittent and AM exposure is titratable. In this context, low-level tonic signalling may help to sustain cell survival between AM doses without precipitating exhaustion; however, data specific to adaptor/modular systems remains limited. The available data suggest that constructs such as PNE-CAR, Uni-CAR, AdCAR, SUPRA-CAR, or the ARC-Sparx system exhibit some tonic signalling owing to intrinsic CAR design, but the quantitative contribution of this signalling to efficacy and toxicity has not been systematically defined. Clarifying the level and quality of tonic signalling and its interaction with AM pharmacokinetics will be essential to optimise persistence while minimising exhaustion.

### 5.3. Adaptor Pharmacokinetics

Another key consideration for modular CAR T cells is the format and the PK of the AM. Given that the majority of modular CARs are antibodies or antibody fragments, the molecular size largely dictates the kinetics: full-sized IgG typically exhibits a half-life of days to weeks whereas fragments below the renal filtration threshold of 60 kDa [99] are cleared rapidly within hours [100]. These PK differences determine whether AMs require continuous infusion or can be administered intermittently in cycles, and how quickly their effects can be attenuated or reversed, which are important factors that directly influence patient safety and quality of life. Ultimately, the decision on the format lies with the development team and should be made considering the manufacturing process and cost, scalability, and finally impact on patient’s quality of life—most people will find treatment in cycles using full-sized antibodies with the possibility of breaks in between more tolerable than a continuous infusion that requires technical assistance and additional devices such as pumps, etc.

### 5.4. Valency

**Figure 2:**
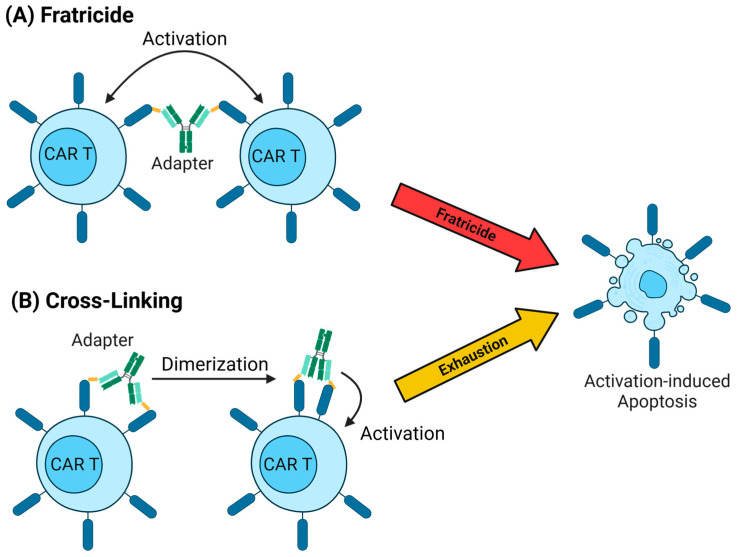
Mechanisms behind (**A**) fratricide and (**B**) cross-linking. Fratricide and cross-linking are two phenomena specific for modular CAR systems stemming from the nature of a two-component system. If the AM has two or more tags, there will always be the possibility of one AM binding multiple CARs; (**A**) if these are on two different cells, both will be activated and subsequently target each other (fratricide) and (**B**) if the CARs are on the same cell, it can lead to dimerization, CAR activation, and exhaustion. Both occurrences will diminish the treatment effect and require consideration and attention when designing and testing the system. Created in BioRender. Joechner, A. (2026) https://BioRender.com/8gkn9q0 (accessed on 27 November 2025).

The valency, the number of CAR binding tags on the AM, is equally critical. Higher valency can increase the potency of the system via increased avidity but also raises the risk for cross-linking, receptor dimerization, and CAR T-cell fratricide. Monovalent AMs recruit only a single CAR molecule at a time, whereas bi- or multivalent AM can engage two or more molecules either on the same cell or neighbouring cells, potentially driving non-specific CAR activation and/or self-targeting of CAR T cells (Figure 2). Size and valency are first-order design levers for AMs that shape dosing strategy, controllability, efficacy, and safety and need to be optimised for each system. Considering these aspects, it is not surprising that the more refined systems such as PNE-CAR, Uni-CAR, or P329G CAR all employ AM tags which are monovalent and genetically fused to enable precise engineering, but the earlier versions such as the FITC- or biotin-CARs relied on chemical and by that stochastic conjugation of the AM and tag.

### 5.5. Immunogenicity

Immunogenicity is a critical consideration in developing immunotherapeutic products; it describes the possibility of a therapy being recognised by the host immune system and leading to the formation of anti-drug antibodies (ADA) [101]. There are many reasons why ADAs are generated but two primary drivers are the species of origin and the molecular format of the agent: fully human antibodies and tag sequences generally carry a lower risk than non-human scaffolds or foreign chemical moieties. In modular CAR systems, where the AM may be administered repeatedly across treatment cycles, minimising immunogenicity is especially important. Humanisation (or de-immunisation) of all exogenous components, particularly the AM and its tag, and any non-human extracellular CAR domains should be prioritised to reduce ADA formation. A good example of this would be the P329G CAR or UniCAR which uses a fully human system, whereas, e.g., the bsCAR is based on a bacterial protein that is not ideal for this purpose.

### 5.6. On-Target, Off-Tumour Toxicity

Two major toxic side effects relating to the systemic immune system activation, CRS and ICANS, have been thoroughly discussed and reviewed elsewhere [102,103]. Here, we focus on on-target, off-tumour (OT-OT) effects, which arise from expression of the chosen TAA on healthy tissues.

CD19-directed therapies for B-cell malignancies predictably cause B-cell aplasia; however, due to immunoglobulin replacement therapy, the impact on quality of life is minimal [104]. By contrast, AML-associated antigens, such as CD33 and CD123, are expressed on hematopoietic stem cells, and sustained depletion may necessitate rescue therapy in the form of hematopoietic stem cell transplantation [105,106,107]. In solid tumours, CAR T cells directed to common tumour targets like GD2, HER2, or B7-H3 encounter OT-OT risks by healthy tissue’s antigen expression: GD2 in peripheral nerves can cause severe neuropathic pain and requires analgesic medication [108]; HER2 expression in lung epithelium confers a risk of pulmonary toxicity, including fatal events [109]; whereas B7-H3 is broadly expressed on solid tumours with limited expression on activated immune cells [110,111]. Modular CAR systems provide several coordinated levers to mitigate OT-OT toxicity by decoupling antigen recognition from T-cell activation, thereby allowing activation thresholds to be tuned, via AM dose and affinity, to preferentially engage tumour cells with high antigen density whilst sparing normal tissues with low-level expression. The system enables plug-and-play logic gating which requires combinatorial antigen expression for CAR activation, improving selectivity, and provides spatiotemporal restriction through step-up dosing or intratumoral delivery. Lastly, the AM clearance supplies an intrinsic off-switch that avoids the need to deplete CAR T cells.

An ideal modular CAR would be non-targeting without the AM which most concepts realise by using non-human tags (PNE, FITC, biotin, or bacterial proteins) but this poses the risk of an immunogenic host reaction against the CAR or AM. Using human sequences, on the other hand, requires careful selection of CAR–AM interaction and rigorous testing for OT-OT toxicity. Prime examples of how to approach these hurdles would be UniCAR and the P329G CAR targeting non-cell surface proteins or specific mutations and the conduit CAR based on an inert germline antibody.

### 5.7. Further Safety and Control Mechanisms

Preclinical and early clinical data suggests that modular CAR platforms generally exhibit fewer proliferation-related toxicities (e.g., CRS and ICANS). Two features likely contribute to this profile: (1) decoupling target recognition from T-cell activation, which introduces a buffer that yields more moderate activation; (2) dose titration of the AM, whose PK translates into graded cytotoxicity. This enables drug-free intervals to mitigate CRS/ICANS, which is a key advantage of modular CAR systems.

Beyond CAR T activation-related safety, the modular design allows for implementation of logic gating by incorporating advances in bi-specific/multi-specific antibodies. Configuring two tumour-antigen recognition domains under rationally designed AND/OR/NOT logic restricts robust CAR T-cell activation to malignant cells only, as demonstrated in the Supra-CAR/Co-LOCKR framework. This capability is particularly important in solid tumours, where a lineage-defining antigen analogous to CD19 is lacking. Dual targeting antigen pairs such as EGFR/cMET, by means of a bi-specific antibody, have shown clinical benefit in solid cancers [112]. In modular CAR systems, on-target/off-tumour effects can be mitigated through rationally designed antigen logic, and the AM-mediated engagement is reversible, further reducing risk.

A further benefit of modularity is the flexibility to use the AM as a delivery vehicle for locally acting payloads. Cytokines (e.g., IL-2 muteins) or other pro-inflammatory agents can be co-delivered to the tumour microenvironment to synergise with recruited CAR T cells, without additional genomic engineering burden on the T cells. This approach also offers superior toxicity control compared with systemic administration of the same payloads.

### 5.8. Future Directions

Modular CAR technology coevolves with advances in the broader CAR T field. As allogeneic manufacturing and in vivo CAR engineering matures and progresses to clinical trials, modular systems are likely to converge with these platforms to deliver true off-the-shelf products or ex vivo expansion-free products that can be retargeted on demand from a single universal cell construct. Beyond the core effector function, rational pairing of CAR T cells with other immune cells through cell engagers can augment cytotoxicity and durability. Within modular architectures, delivery of the AM can be configured as cell-based secretion or as exogenous dosing. The former creates “micro pharmacies” in which engineered cells provide local adaptor production in the tumour microenvironment [113,114], whereas the latter enables precise PK and rapid reversibility.

A more controllable modular CAR system should use adaptive dosing guided by biomarkers such as circulating adaptor levels, target occupancy, and clonal expansion kinetics to individualise exposure, maximise efficacy, and limit toxicity. These control features are naturally synergistic with checkpoint inhibitors, which can relieve exhaustion at peaks of adaptor-driven synapse formation, and with T-cell engagers that recruit bystander T cells to create an immune-permissive niche for CAR T cells. In parallel, programming cells to remodel the tumour microenvironment through targeted secretion of cytokines, chemokines, or matrix-modifying enzymes can improve trafficking, persistence, and antigen access.

Because modular platforms combine a living cell therapy with a drug-like adaptor, regulatory pathways will need to support cross-label instructions for use and allow post-approval changes to one component without revalidating the other. Taken together, modular off-the-shelf CAR regimens can move beyond one-and-done cell therapy towards a precise, retargetable pharmacology that scales into routine clinical practice.

## 6. Conclusions

In summary, the ideal modular CAR incorporates fully human CAR and tag sequences with moderate affinity (neither too high nor too low) and low tonic signalling, thereby limiting immunogenicity whilst maximising the control, safety, and longevity of CAR T cells. Toxicity will remain largely determined by antigen selection, but the modular format allows rapid exchange or even simultaneous use of targets, reducing adverse effects and enabling patient-specific tuning of anticancer therapy. These features are mostly reflected in the systems currently undergoing clinical evaluation (PNE-CAR, Uni-CAR, P329G CAR, etc.) and the outcome of these trials will direct future developments. Leveraging these features, modular CAR T cells can redefine cellular therapy as an effective, safe, and individualised approach in which clinicians respond swiftly to evolving tumour biology and, ultimately, improve patient outcomes.

## Figures and Tables

**Table 1:** Landscape of modular CAR constructs in 2025.

Coupling Technology	Platform	CAR Target (Clone)	Adaptor Format	Adaptor Tag	Tag Valency	References
Peptide Tags	Uni-CAR	La/SS-B (5B9)	scFv, nanobody	La/SS-B-derived peptide	Monovalent	[15,33]
	PNE-CAR	PNE GCN4 (52SR4)	Fab	GCN4-derived peptide	Monovalent	[16]
	AdCAR v2	P2m2 (MB196-C1)	Fab	FGFR2-derived peptide	Monovalent	[34]
	cp-Fab/CAR	PNE GCN4 (52SR4)	Fab–folate conjugate	GCN4-derived peptide	Monovalent	[35]
	SAR T	EGFR/Cripto-1 scFv	Bi-specific mAb	EGFR/Cripto-1 Fab or scFv	Bivalent	[36]
	RevCAR	5B9/7B6 scFv	scFv	5B9/7B6 scFv	Monovalent	[37]
	Fabrack-CAR	Meditope mAb or Fab	Fab or mAb	Meditope binding site	Mono- or bivalent	[25]
	Conduit CAR	Unknown (3-23/B3) CD19 (FMC63)	Bi-specific mAb	G4S-targeting Fab or scFv	Bivalent	[38]
	TRUE CAR T	EGFRvIII	PEG–lipid conjugate	EGFRvIII peptide	Monovalent	[39]
Chemical Tags	FITC CAR	FITC (4m5.3)	Fab	FITC	Mono- or bivalent	[17]
	FITC CAR	FITC (4m5.3)	Small molecule	FITC	Monovalent	[18]
	FITC CAR	FITC (E2)	Small molecule	FITC	Monovalent	[40]
	Amph-FITC CAR	FITC (4m5.3)	PEG–lipid conjugate	FITC	Monovalent	[41]
	AdCAR	Biotin-LLE (mBio3)	mAb	Biotin-LLE	Multivalent	[19]
	mSA2 CAR	Biotin	mAb	Biotin	Multivalent	[20]
	BBIR CAR	Biotin	scFv, mAb	Biotin	Multivalent	[21]
	BsAb-IR CAR	FRα mAb	Two conjugated mAbs	FRα mAb	Bivalent	[42]
Antibody domain recognition	CD16 CAR	Fc part of mAb	mAb	Fc part of mAb	Bivalent	[43]
	ACTR (CD16 CAR)	Fc part of mAb	mAb	Fc part of mAb	Bivalent	[22]
	CD16 CAR	Fc part of mAb	mAb	Fc part of mAb	Bivalent	[44]
	CD16 CAR	Fc part of mAb	mAb	Fc part of mAb	Bivalent	[23]
	P329G CAR	P329G mutation	Fc silenced mAb	P329G mutation of Fc part	Monovalent	[24]
	GA1 CAR	Fab C_L_ domain (GA1)	Fab	C_L_ domain of Fab	Monovalent	[26]
	CD19 CAR engager	CD19 (FMC63)	scFv	CD19-ECD	Monovalent	[45]
Covalent bond	OmniCAR	SpyTag	DARPin or mAb	SpyTag	Mono- or bivalent	[29]
	MU-CAR	GvOptiTag	scFv	GvOptiTag	Monovalent	[28]
	BsCAR	Barnase	DARPin	Barnase	Monovalent	[27]
	SNAP-CAR	Benzylguanine	mAb	Benzylguanine	Multivalent	[46]
Engineered protein pairs	Supra-CAR	Cognate leucine zipper	scFv	Cognate leucine zipper	Monovalent	[30]
	Co-LOCKR	Bim “latch”	DARPin	“Cage” and “key” protein	Monovalent	[31]
	ConvertibleCAR	ULBP2-S3	mAb	U2S3 peptide	Bivalent	[32]
	ARC-SparX	AFP domain III	D-domain	AFP-derived peptide	Mono- or bivalent	[47]

**Table 2:** Clinical trials investigating modular CAR T-cell therapies. N/A: information not publicly disclosed or reported.

Platforms	Coupling Technology	Target Antigen and Disease Entity	Clinical Trial	Status	Results	References
Uni-CAR	scFv binds La/SS-B-derived peptide	CD123-positive AML	NCT04230265	Phase I, active, not recruiting	No DLT, clinical response in all patients, ORR 53% in first 19 patients	[89]
		PSMA-positive prostate cancer	NCT04633148	Phase I, terminated	N/A	
RevCAR	La/SS-B peptide binds scFv	CD123-positive AML	NCT05949125	Phase I, recruiting	N/A	
PNE-CAR	scFv binds GCN4-derived PNE	CD19-positive B-cell malignancy	NCT04450069	Phase I, completed	Safe and well tolerated, clinical response, ORR 78% and CR 67%	[55,56]
		Her2-positive breast cancer	NCT06878248	Phase I, recruiting	N/A	
		CD19, autoimmune diseases	NCT06913608	Phase I, not yet recruiting	N/A	
ACTR	CD16 ECD binding IgG Fc domain	Her2-positive advanced malignancies	NCT03680560	Phase I, terminated	N/A	
		CD20-positive B-cell lymphoma	NCT02776813	Phase I, completed	CRS and neurotoxicity reportedORR 50%	[73]
		CD20-positive B-cell lymphoma	NCT03189836	Phase I, terminated	well tolerated, ORR 56%	[72]
P329G CAR	scFv binds P329G mutated Fc domain	CLDN18.2-positive solid tumours	NCT05199519	Phase I, completed	Manageable safety profile and preliminary efficacy	[77]
		BCMA-positive multiple myeloma	NCT05270928	Phase I, completed	No DLT, PR 50%	[79]
		BCMA-positive multiple myeloma	NCT05266768	Phase I, unknown	No DLT, PR 40%	[79]
CD19 CAR engager	scFv binds CD19 ECD	B-cell malignancies post CD19 CAR T-cell therapy	NCT06045910	Phase I/II, recruiting	N/A	
convertibleCAR	iNKG2D ECD binds ULBP2-S3	CD20-positive B-cell lymphoma	NCT06248086	Phase I, terminated	N/A	
ARC-SparX	scFv binds AFP-derived peptide	CD123-positive AML or MDS	NCT05457010	Phase I, recruiting	N/A	

## Data Availability

No new data were created or analyzed in this study. Data sharing is not applicable to this article.

## Glossary

AA	Amino acid
ACTR	Antibody coupled T-cell receptor
ALL	Acute lymphoid leukaemia
AM	Adaptor molecule
AML	Acute myeloid leukaemia
ADA	Anti-drug-antibodies
AFP	Alpha-fetoprotein
ARC	Antigen–receptor complex
BBIR	Biotin-binding immune receptor
BCL-2	B-cell lymphoma 2
BG	Benzylguanine
Bim	Bcl2-interacting mediator of cell death
BsAb-IR	BsAb-binding immune receptor
BsCAR	Barnase/barstar-based CAR
CAR	Chimeric antigen receptor
CA IX	Carbonic anhydrase IX
C_L_	Constant light chain
CRS	Cytokine release syndrome
DARPins	Designed ankyrin repeat proteins
DLT	Dose-limiting toxicity
EC_50_	Effective concentration
EGFR	Epidermal growth factor receptor
F-AgNPs	Fusogenic antigen loading nanoparticles
FGFR2	Fibroblast growth factor receptor 2
FITC	Fluorescein isothiocyanate
FOLR1/FRα	Folate receptor 1/alpha
Gv	GvOptiTag
iNKG2D	Inert NKG2D variant
ICANS	Immune effector cell-associated neurotoxicity syndrome
LLE	Linker–label epitope
MDS	Myelodysplastic syndrome
MM	Multiple myeloma
mSA2	Monomeric streptavidin 2
MU-CAR-T	Modular universal CAR T
ORR	Overall response rate
PR	Partial response
sCAR-T	Switchable CAR-T-cell
Sd	SDCatcher
TAA	Tumour-associated antigen
TMEFF2	Tomoregulin-2
OT-OT	On-target–off-tumour
O6-BG	O6-benzylguanine
PNE	Peptide neo epitope
PSCA	Prostate stem cell antigen
PSMA	Prostate-specific membrane antigen
